# Supplementary material for: Pili allow dominant marine cyanobacteria to avoid sinking and evade predation
Source: Nat Commun. 2021 Mar 25;12:1857. doi: 10.1038/s41467-021-22152-w (PMC7994388; doi:10.1038/s41467-021-22152-w)
Supplement: Supplementary file 3 — Description of Additional Supplementary Files [file 41467_2021_22152_MOESM3_ESM.pdf]

**Description of Additional Supplementary Files**

File Name: Supplementary Data 1
Description: Presence of structural pilus genes in cultured picocyanobacterial isolates and SAGs

File Name: Supplementary Data 2
Description: Sinking rate of wild type and pili mutant strains of Synechococcus sp. WH7803

File Name: Supplementary Data 3
Description: Cellular proteomes and exoproteome quantification and comparative proteomic analysis of wild type and pili mutant Synechococcus sp. WH7803 strains.

File Name: Supplementary Data 4
Description: Exoproteome quantification and comparative proteomic analysis of Prochlorococcus sp. MIT9313 under different conditions.

File Name: Supplementary Data 5
Description: Exoproteome quantification and comparative proteomic analysis of Synechococcus sp. WH7803 when grown under different nutrient starvation conditions.

File Name: Supplementary Data 6
Description: Exoproteome quantification and comparative proteomic analysis of Synechococcus sp. WH7803 when grown under different nutrient concentration.

File Name: Supplementary Data 7
Description: Exoproteome quantification and comparative proteomic analysis of Synechococcus sp. WH7803 when grown under different light regimes.

File Name: Supplementary Movie 1
Description: Suspended wild type cells of Synechococcus sp. WH7803.

File Name: Supplementary Movie 2
Description: Suspended pili mutant cells of Synechococcus sp. WH7803.

File Name: Supplementary Movie 3
Description: . Tracked suspended wild type cell of Synechococcus sp. WH7803.

File Name: Supplementary Movie 4
Description: Tracked suspended pili mutant cell of Synechococcus sp. WH7803.
