# Supplementary material for: Pili allow dominant marine cyanobacteria to avoid sinking and evade predation
Source: Nat Commun. 2021 Mar 25;12:1857. doi: 10.1038/s41467-021-22152-w (PMC7994388; doi:10.1038/s41467-021-22152-w)
Supplement: Supplementary file 15 — Reporting Summary [file 41467_2021_22152_MOESM15_ESM.pdf]

nature research

Corresponding author(s): Joseph A. CHRISTIE-OLEZA

Last updated by author(s): Feb 24, 2021

Reporting Summary

Nature Research wishes to improve the reproducibility of the work that we publish. This form provides structure for consistency and transparency in reporting. For further information on Nature Research policies, see our Editorial Policies and the Editorial Policy Checklist.

Statistics

For all statistical analyses, confirm that the following items are present in the figure legend, table legend, main text, or Methods section.

n/a	Confirmed	
☐	☒	The exact sample size (*n*) for each experimental group/condition, given as a discrete number and unit of measurement
☐	☒	A statement on whether measurements were taken from distinct samples or whether the same sample was measured repeatedly
☐	☒	The statistical test(s) used AND whether they are one- or two-sided<br>*Only common tests should be described solely by name; describe more complex techniques in the Methods section.*
☒	☐	A description of all covariates tested
☒	☐	A description of any assumptions or corrections, such as tests of normality and adjustment for multiple comparisons
☐	☒	A full description of the statistical parameters including central tendency (e.g. means) or other basic estimates (e.g. regression coefficient) AND variation (e.g. standard deviation) or associated estimates of uncertainty (e.g. confidence intervals)
☐	☒	For null hypothesis testing, the test statistic (e.g. *F*, *t*, *r*) with confidence intervals, effect sizes, degrees of freedom and *P* value noted<br>*Give P values as exact values whenever suitable.*
☒	☐	For Bayesian analysis, information on the choice of priors and Markov chain Monte Carlo settings
☒	☐	For hierarchical and complex designs, identification of the appropriate level for tests and full reporting of outcomes
☒	☐	Estimates of effect sizes (e.g. Cohen's *d*, Pearson's *r*), indicating how they were calculated

Our web collection on statistics for biologists contains articles on many of the points above.

Software and code

Policy information about availability of computer code

Data collection	Confocal microscope images were collected using Leica application suite (Leica Microsystems). Sinking cell images were operated with FlyCapture2 (FLIR Systems UK). Flow cytometer (BD Fortessa) was operated by BD FACSDiva™ Software.
Data analysis	Cells (length and width) and pili (length and diameter) were measured using ImageJ (version 1.53a). Sedimentation velocities were calculated in MATLAB 2019a using particle tracking code from ref 42. Mass spectra were identified and quantified using the LFQ default parameters in MaxQuant v1.6.10.43. Comparative proteomic analyses were performed using Perseus v1.6.2.2. Genome were re-annotated using default parameters in PROKKA vs 1.7. HMM profile was performed in Unipro UGENE vs 33 implemented with the hmmbuild programme from HMM3 using the default parameters. HMM profile was used to search the TARA oceans metagenomes and metatranscriptomes via the functions offered in the Ocean Gene Atlas portal (ref 30). 3D protein structures were performed using the I-TASSER server's default settings (ref 51).

For manuscripts utilizing custom algorithms or software that are central to the research but not yet described in published literature, software must be made available to editors and reviewers. We strongly encourage code deposition in a community repository (e.g. GitHub). See the Nature Research guidelines for submitting code & software for further information.

Data

Policy information about availability of data

All manuscripts must include a data availability statement. This statement should provide the following information, where applicable:

- Accession codes, unique identifiers, or web links for publicly available datasets
- A list of figures that have associated raw data
- A description of any restrictions on data availability

All detailed methods and data is available as supplementary information and data. Source data for Figs 3 and S6 are provided as a Source Data file. The RAW mass spectrometry proteomics data have been deposited in the ProteomeXchange Consortium via the PRIDE partner repository with the dataset identifiers: PXD018394,

PXD018395, PXD018396, PXD018524 and PXD019315. Cultured marine picocyanobacterial genomes were downloaded from the Cyanorak database v2.1 (ref. 23). Picocyanobacterial SAGs were downloaded from ref. 29 (https://figshare.com/collections/Single_cell_genomes_of_i_Prochlorococcus_i_i_Synechococcus_i_and_sympatric_microbes_from_diverse_marine_environments/4037048). TARA metagenomes and metatranscriptomes were analysed using the Ocean Gene Atlas portal (ref. 30).

Field-specific reporting

Please select the one below that is the best fit for your research. If you are not sure, read the appropriate sections before making your selection.

- [x] Life sciences
- [ ] Behavioural & social sciences
- [ ] Ecological, evolutionary & environmental sciences

For a reference copy of the document with all sections, see nature.com/documents/nr-reporting-summary-flat.pdf

Life sciences study design

All studies must disclose on these points even when the disclosure is negative.

Sample size	Sample size was set to the standard 3 independent biological replicates per treatment. This was sufficient as shown by the clear differences between treatments and small deviation between replicates.
Data exclusions	From all single-cell assembled genomes (SAGs) we downloaded from Berube et al 2018 we decided to take a minimum degree of SAG completeness equal or above 75% as we believed anything below this value would distort the results due to the lack of gene completeness.
Replication	All experiments were performed with 3 independent biological replicates except for the proteomic analysis of the exoproteomes of Prochlorococcus sp. MIT9313 which were performed in duplicates as this was just to confirm the presence/absence of pili in the cultures.
Randomization	Cultures were always initiated from one single culture and all cultures were randomly distributed in the incubator when monitoring growth to avoid covariates.
Blinding	Blinding was not relevant to our study because there was no possible subjective interpretation of the data (e.g. cell sinking, statistical analysis of the proteomics data, differences between wild type and mutant strain).

Reporting for specific materials, systems and methods

We require information from authors about some types of materials, experimental systems and methods used in many studies. Here, indicate whether each material, system or method listed is relevant to your study. If you are not sure if a list item applies to your research, read the appropriate section before selecting a response.

Materials & experimental systems

n/a	Involved in the study	
x		Antibodies
x		Eukaryotic cell lines
x		Palaeontology and archaeology
x		Animals and other organisms
x		Human research participants
x		Clinical data
x		Dual use research of concern

Methods

n/a	Involved in the study	
x		ChIP-seq
	x	Flow cytometry
x		MRI-based neuroimaging

Flow Cytometry

Plots

Confirm that:

- [x] The axis labels state the marker and fluorochrome used (e.g. CD4-FITC).
- [x] The axis scales are clearly visible. Include numbers along axes only for bottom left plot of group (a 'group' is an analysis of identical markers).
- [x] All plots are contour plots with outliers or pseudocolor plots.
- [x] A numerical value for number of cells or percentage (with statistics) is provided.

Methodology

Sample preparation	Auto-fluorescent cyanobacteria were diluted to 10E5 - 10E6 cell/ml using sterile seawater and quantified by including reference beads (Fluorescent Nile Red beads; ~2 µm spherical particles; Spherotech).

Instrument	Becton Dickinson Fortessa Flow Cytometer
Software	BD FACSDiva™ Software
Cell population abundance	Cell abundance was evaluated by the number of cells in the corresponding gate (FSC and PE) when 100 bead particles had been counted. Cell abundance was calculated accordingly to the dilution of cells and beads.
Gating strategy	Staining was not necessary. Gating was established from samples where only beads and cyanobacterial cells were present.

☒ Tick this box to confirm that a figure exemplifying the gating strategy is provided in the Supplementary Information.
